# Effect of Silica Embedding on the Structure, Morphology and Magnetic Behavior of (Zn_0.6_Mn_0.4_Fe_2_O_4_)_δ_/(SiO_2_)_(100−δ)_ Nanoparticles

**DOI:** 10.3390/nano11092232

**Published:** 2021-08-29

**Authors:** Thomas Dippong, Iosif Grigore Deac, Oana Cadar, Erika Andrea Levei

**Affiliations:** 1 Faculty of Science, Technical University of Cluj-Napoca, 76 Victoriei Street, 430122 Baia Mare, Romania; 2 Faculty of Physics, Babes-Bolyai University, 1 Kogalniceanu Street, 400084 Cluj-Napoca, Romania; iosif.deac@phys.ubbcluj.ro; 3 INCDO-INOE 2000, Research Institute for Analytical Instrumentation, 67 Donath Street, 400293 Cluj-Napoca, Romania; oana.cadar@icia.ro (O.C.); erika.levei@icia.ro (E.A.L.)

**Keywords:** silica matrix, sol-gel synthesis, zinc-manganese ferrite, magnetic properties

## Abstract

The effect of SiO_2_ embedding on the obtaining of single-phase ferrites, as well as on the structure, morphology and magnetic properties of (Zn_0.6_Mn_0.4_Fe_2_O_4_)_δ_(SiO_2_)_100−δ_ (δ = 0–100%) nanoparticles (NPs) synthesized by sol-gel method was assessed. The phase composition and crystallite size were investigated by X-ray diffraction (XRD), the chemical transformations were monitored by Fourier transform infrared (FT-IR) spectroscopy, while the morphology of the NPs by transmission electron microscopy (TEM). The average crystallite size was 5.3–27.0 nm at 400 °C, 13.7–31.1 nm at 700 °C and 33.4–49.1 nm at 1100 °C. The evolution of the saturation magnetization, coercivity and magnetic anisotropy as a function of the crystallite sizes were studied by vibrating sample magnetometry (VSM) technique. As expected, the SiO_2_ matrix shows diamagnetic behavior accompanied by the accidentally contribution of a small percent of ferromagnetic impurities. The Zn_0.6_Mn_0.4_Fe_2_O_4_ embedded in SiO_2_ exhibits superparamagnetic-like behavior, whereas the unembedded Zn_0.6_Mn_0.4_Fe_2_O_4_ behaves like a high-quality ferrimagnet. The preparation route has a significant effect on the particle sizes, which strongly influences the magnetic behavior of the NPs.

## 1. Introduction

Zinc ferrite (ZnFe_2_O_4_) has a normal spinel structure and remarkable magnetic, electrical, electrochemical and sensing properties, making it suitable for a wide-range of applications [1,2,3,4]. Its excellent magnetization is attributed to the inversion of Fe^3+^ and Zn^2+^ ions between tetrahedral (A) and octahedral (B) sites [4]. Manganese ferrite (MnFe_2_O_4_) has a partially inverse spinel structure and numerous applications due to its tunable magnetic properties, small-sized particles, possibility to be controlled by an external magnetic field, easy synthesis process and biocompatibility. It is also an inorganic heat-resistant, non-corrosive, non-toxic and environmentally friendly material with coloristic properties [5,6,7].

Due to their unique properties, the Mn-Zn ferrites are widely used in many fields such as medicine, environmental depollution, energy storage, gas sensors and photocatalysis [8,9,10]. The use of nanoferrites in medicine is possible due to their ability to locally heat up the tissues under an external variable magnetic field as a consequence of thermal losses. The high sensitivity to oxidation and cytotoxicity of pure metal particles makes them inadequate for medical applications, while iron oxide nanoparticles are promising candidates, due to their biocompatibility, especially if they are covered with inorganic or organic biocompatible coatings (i.e., alcohol, esters, SiO_2_) [11]. Recently, due to their low toxicity, Mn-Zn ferrites become a center of attraction for hyperthermia, electrical, electronic and telecommunication devices [12,13]. Cubic spinel Mn-Zn ferrites belonging to soft ferrites are interesting magnetic materials due to the low core loss, corrosion resistance, high saturation magnetization (*M_S_*), high magnetic permeability and low eddy loss [10,12].

The properties of spinel ferrites can be easily tuned and controlled as they depend on the composition and particle size distribution. The oxidation state of cations and their distribution in the spinel structure also impact the magnetic behavior of ferrites [12,14]. Mixed Mn-Zn ferrites have the spinel structure with Fe^3+^ ions occupying tetrahedral (A) and octahedral (B) sites, and the Mn^2+^ and Zn^2+^ ions occupying A sites [15]. The variation in this “normal” arrangement leads to an inverted spinel structure, with Fe^3+^ ions in A sites, and Mn^2+^ and Zn^2+^ ions in B sites. Additionally, the ferromagnetic arrangement of spinel structure with the magnetic moment in the A site will align antiparallel to any external magnetic field. Therefore, the overall magnetic behavior of Mn-Zn ferrites is associated to the particle morphology and cation distribution in the crystalline structure [8]. The structural, magnetic and electric properties may be changed by introducing various cations into the spinel structure [15].

The Mn-Zn ferrites were produced by microwave, co-precipitation, pyrolysis, decomposition, sol-gel, auto-combustion, hydrothermal and solid-state techniques. The synthesis route plays a key role in tailoring the structure, morphology and properties of the NPs [7,8,9,10]. Generally, the chemical methods give fine grained microstructure, convoyed by long reaction time and post-synthesis thermal treatment; however, often poor crystallinity and broad particle size distribution can alter the desired properties [9,12]. The sol-gel route is a common way to prepare ferrite NPs due to its simplicity, low cost and good control over the structure and properties. Encapsulating ferrite NPs into solid silica (SiO_2_) allows the control of the particle growth, minimization of particle agglomeration, as well as the enhancement of their magnetic guidability and biocompatibility [5].

This paper aims to investigate the structure, morphology and magnetic properties of (Zn_0.6_Mn_0.4_Fe_2_O_4_)_δ_(SiO_2_)_100−δ_ NPs produced by sol-gel route as well as the effect of various factors such as the ferrite type, embedding material, synthesis method, stoichiometric composition and calcination temperature.

## 2. Materials and Methods

All chemical reagents, of analytical grade, were purchase from Merck (Darmstadt, Germany) and used without further purification. (Zn_0.6_Mn_0.4_Fe_2_O_4_)_δ_(SiO_2_)_100−δ_ (δ = 0–100%) NPs were synthesized by sol-gel method. Zinc nitrate (Zn(NO_3_)_2_·6H_2_O), manganese nitrate (Mn(NO_3_)_2_·3H_2_O) and ferric nitrate (Fe(NO_3_)_3_·9H_2_O) were dissolved in 1,4-butanediol (BD) in a molar ratio of 0.6:0.4:2:8. To the nitrate-BD mixture, an ethanolic solution of tetraethyl orthosilicate (TEOS) acidified with nitric acid (pH = 2) in NO_3_^−^:TEOS molar ratio of 0:2 (δ = 0%), 0.5:1.5 (δ = 25%), 1:1 (δ = 50%), 1.5:0.5 (δ = 75%) and 2:0 (δ = 100%) was added dropwise with continuous stirring, at room temperature. The obtained mixture was exposed to open air for gelation (8 weeks). The glassy gels were grinded and dried at 300 °C for 4 h, then calcined in air, at 400, 700 and 1100 °C for 5 h in a LT9 muffle furnace (Nabertherm, Lilienthal, Germany).

The morphology of NPs was investigated on dried suspensions of NPs onto carbon-coated copper grids using a Hitachi HD-2700 (Hitachi, Tokyo, Japan) transmission electron microscope (TEM) equipped with digital image recording system. The effect of the ferrite content embedded in the amorphous SiO_2_ matrix on structural properties was investigated by X-ray diffraction pattern recorded at room temperature, using a D8 Advance (Bruker, Karlsruhe, Germany) diffractometer, operating at 40 kV and 40 mA with CuKα radiation (λ = 1.54060 Å). The formation of the ferrite and SiO_2_ matrix was monitored using a Spectrum BX II (Perkin Elmer, Waltham, MA, USA) Fourier-transform infrared spectrometer on KBr pellets containing 1% sample. A cryogen free VSM magnetometer (Cryogenic Ltd., London, UK) was used for magnetic measurements. The saturation magnetization was measured in high magnetic field up to 10 T, while the magnetic hysteresis loops were performed between −2 to 2 T, at 300 K on samples incorporated in an epoxy resin. The particles were not aligned in an applied magnetic field.

## 3. Results and Discussion

**Figure 1:**
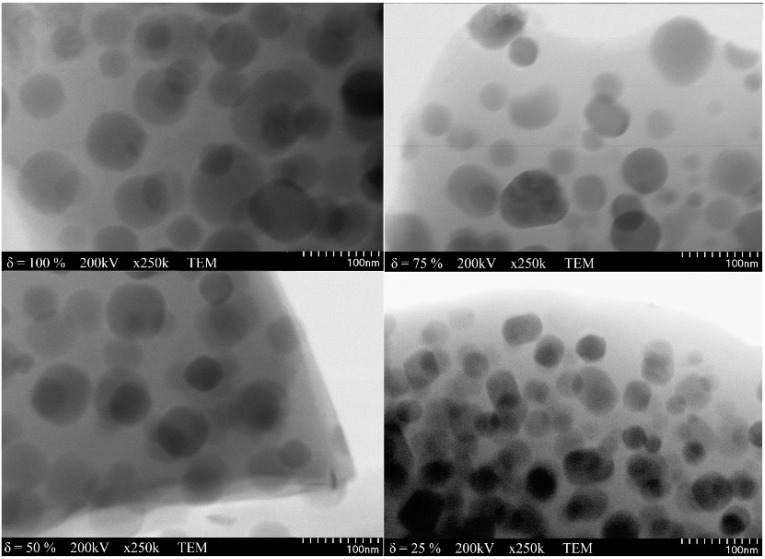
TEM images of (Zn_0.6_Mn_0.4_Fe_2_O_4_)_δ_(SiO_2_)_100−δ_ NPs calcined at 1100 °C.

Figure 1 shows the distribution, shape and size of (Zn_0.6_Mn_0.4_Fe_2_O_4_)_δ_(SiO_2_)_100−δ_ NPs (δ = 25–100%) calcined at 1100 °C. The TEM images of SiO_2_ (δ = 0%) does not allow the identification of the SiO_2_ network. In case of samples calcined at low temperatures the TEM images are blurry with particles that are not clearly separated. The TEM images show irregular, spongy aggregates of spherical, large (50 nm for δ = 100%, 41 nm for δ = 75%) NPs in case of high ferrite content and small (34 nm for δ = 25%, 35 nm for δ = 50%) NPs in case of high SiO_2_ content. The different size and morphology of NPs could be the consequence of the different kinetics of metal oxides formation reaction and of the different particle growth rate following volume expansion and supersaturation reduction [13,14,15].

**Figure 2:**
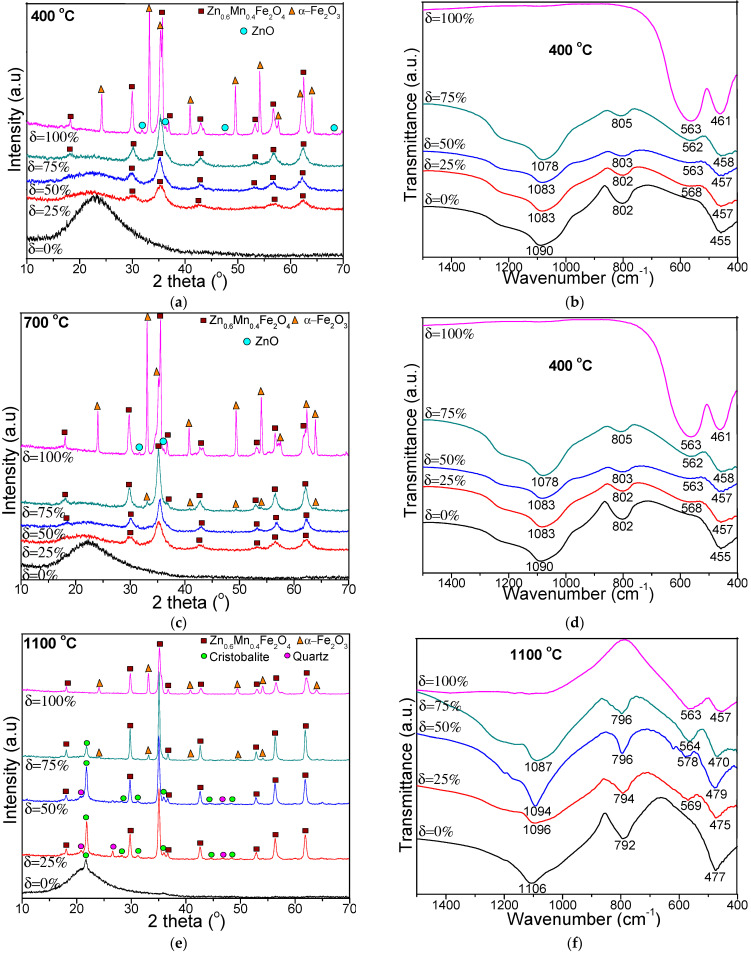
XRD patterns (**a**,**c**,**e**) and FT-IR spectra (**b**,**d**,**f**) of (Zn_0.6_Mn_0.4_Fe_2_O_4_)_δ_(SiO_2_)_100−δ_ NPs calcined at 400, 700 and 1100 °C.

Figure 2 shows the XRD patterns and FT-IR spectra of NPs calcined at 400, 700 and 1100 °C. The degree of crystallinity was estimated as the ratio between the area of all diffraction peaks and the total area of diffraction peaks and amorphous halo [13,16]. In case of δ = 0%, the formation of amorphous SiO_2_ matrix is confirmed by the broad halo in the 2θ range of 15–30°, at all temperatures. At low calcination temperatures (400 and 700 °C), crystalline Zn_0.6_Mn_0.4_Fe_2_O_4_ (ZnFe_2_O_4_ (JCPDS card 16-6205 [17]) and MnFe_2_O_4_ (JCPDS card 10-0319 [17]) phases with cubic spinel structure belonging to *Fd3m* group are remarked. At 400 °C, in case of the NPs with δ = 25% and 50%, the amorphous halo in the 2θ range of 15–30° is also remarked, but much flatten than in case of δ = 0%.

At 700 °C, at higher ferrite content (δ = 75%), the main Zn_0.6_Mn_0.4_Fe_2_O_4_ phase is accompanied by α-Fe_2_O_3_ secondary phase (JCPDS card 87-1164) [17]) most probably due to the fact that ferrite is only partially embedded in the SiO_2_ matrix, as a consequence of the low SiO_2_ content. The presence of α-Fe_2_O_3_ might be also attributed to the insufficient calcination temperature or time needed to obtain pure crystalline Zn_0.6_Mn_0.4_Fe_2_O_4_ phase [18]. In case of Zn_0.6_Mn_0.4_Fe_2_O_4_ (δ = 100%), well-crystallized Zn_0.6_Mn_0.4_Fe_2_O_4_ and α-Fe_2_O_3_ phases together with low-crystallized ZnO phase (JCPDS card 36-1451) [17]) is observed. Consequently, it is possible that the SiO_2_ matrix may favor the formation of single-crystalline Zn_0.6_Mn_0.4_Fe_2_O_4_ phase.

At 1100 °C, in case of δ = 0%, the broad halo belonging to the SiO_2_ matrix displays a sharp peak attributed to cristobalite (JCPDS card 89-3434 [17]), which leads to the assumption that the SiO_2_ matrix is partially crystalline, in the absence of ferrite. In case of NP with δ = 25% and 50%, beside the main Zn_0.6_Mn_0.4_Fe_2_O_4_ phase, the secondary phases belonging to the SiO_2_ matrix (cristobalite and quartz, JCPDS card 71-0785 [17]) is also remarked. At higher ferrite content (δ = 75%), the crystalline quartz phase disappears, the cristobalite content is reduced and the main Zn_0.6_Mn_0.4_Fe_2_O_4_ phase is accompanied by the low-crystallized α-Fe_2_O_3_ phase. In case of Zn_0.6_Mn_0.4_Fe_2_O_4_ (δ = 100%) calcined at 1100 °C, the degree of crystallinity of α-Fe_2_O_3_ decreases, as suggested the less intense diffraction peaks compared to the NPs calcined at 400 and 700 °C. The peaks corresponding to ferrite embedded in SiO_2_ matrix, become more intense at 1100 °C, indicating high degree of crystallinity, high crystallite size (because coalescence process) and high nucleation rate (due to the small growth rate and homogenously distributed nanoparticles) and low effect of inert surface layer of the crystals [13,19]. Furthermore, the position of the highest peak is shifted towards higher angles with increasing Zn_0.6_Mn_0.4_Fe_2_O_4_ content in the SiO_2_ amorphous matrix.

The average crystallite size calculated using the Debye-Scherrer’s equation [13,16] considering the highest intensity peak (311) and the quantitative phase analysis using reference intensity ratio method of the NPs are presented in Table 1. The crystalline SiO_2%_ represents the sum of SiO_2_-cristobalite % and SiO_2_-quartz %. The content of crystalline SiO_2_ decreases, while that of α-Fe_2_O_3_ increases with the increase of ferrite content embedded in the SiO_2_ matrix. In addition, the average crystallites size increases from 5.3 to 49.1 nm with the increase of calcination temperature and ferrite content embedded in SiO_2_ [20,21,22]. The peak intensity also increases with the calcination temperature, leading to enhanced crystallinity of the Zn_0.6_Mn_0.4_Fe_2_O_4_. In addition, the crystalline volume to surface ratio is increasing alongside calcination temperature, as showed in the TEM data, due to particle size expansion [21].

At all temperatures, the FT-IR spectra of NPs with δ = 25–100% show the absorption bands of Zn-O and Mn-O bonds stretching vibration at 562–578 cm^−1^ and of Fe–O bonds vibration at 457–479 cm^−1^ [20]. The intensity of the vibration band at 562–578 cm^−1^ increases with the increase of calcination temperature, most probably due to the increase of the ferrite’s crystallinity, as ferrites acts as continuously bonded crystals with atoms linked to all nearest neighbors by equivalent ionic, covalent or van der Waals forces [21,23,24]. The increase of calcination temperature determines a small shift of the vibration band due to the changes in the ion’s distribution between A and B sites [23,24,25,26]. The specific bands of the SiO_2_ matrix were identified in the FT-IR spectra of NPs with δ = 0–75%: 1078–1106 cm^−1^ with a shoulder around 1200 cm^−1^ assigned to vibration of Si–O–Si chains, 792–809 cm^−1^ assigned to the vibrations of SiO_4_ tetrahedron and 457–479 cm^−1^ assigned to the Si-O bond vibration that is overlapping the band of Fe–O vibration [13,19]. The low polycondensation degree of the SiO_2_ network is suggested by the high intensity of these bands.

**Figure 3:**
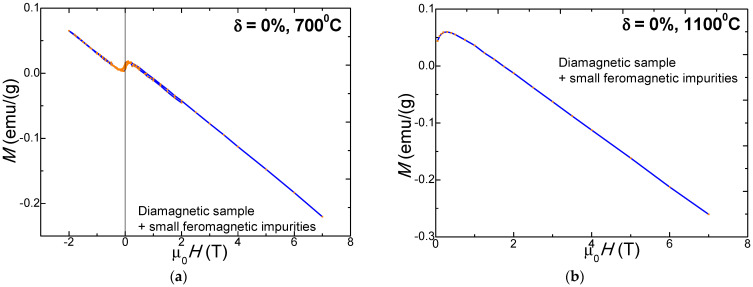
Diamagnetic properties of the SiO_2_ matrix (δ = 0%) for the samples calcined at 700 °C (**a**) and 1100 °C (**b**).

The SiO_2_ matrix (δ = 0%) calcined at 700 and 1100 °C displays diamagnetic behavior at high magnetic fields, while at low magnetic fields it shows the signature of the presence of a low concentration of some ferromagnetic impurities (Figure 3).

**Figure 4:**
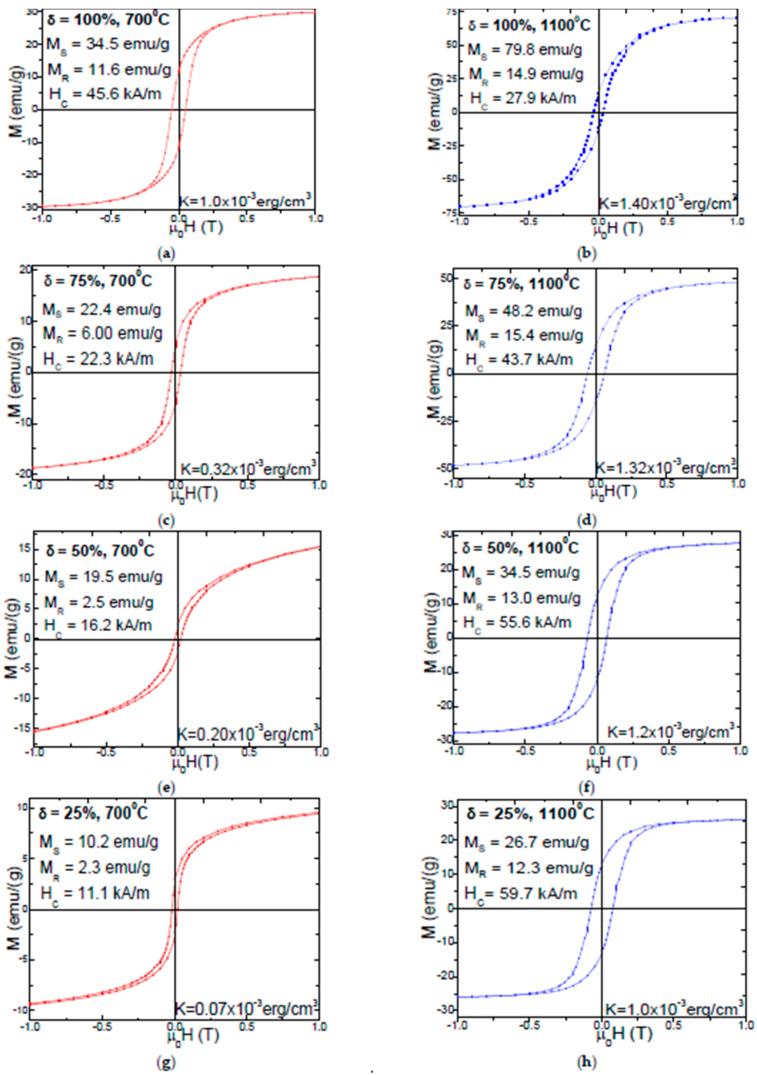
Magnetic hysteresis loops of (Zn_0.6_Mn_0.4_Fe_2_O_4_)_δ_(SiO_2_)_100−δ_ (δ = 100% (**a**,**b**), 75% (**c**,**d**), 50% (**e**,**f**) and 25% (**g**,**h**)) NPs calcined at 700 and 1100 °C.

The room temperature magnetic hysteresis loops, the saturation magnetization (*M_S_*), remanent magnetization (*M_R_*), coercive field (*H_c_*) values of (Zn_0.6_Mn_0.4_Fe_2_O_4_)_δ_(SiO_2_)_100−δ_ (δ = 25–100%) NPs calcined at 700 and 1100 °C are shown in Figure 4.

Typical ‘S’ shape hysteresis loops are obtained for all the investigated NPs, indicating their soft magnetic behavior. For the NPs calcined at low temperature (400 °C), the magnetic parameters values are very small, making difficult to quantify the effect of the heat treatment on the sample magnetic properties. Contrarily, the hysteresis loops show saturation under the same magnetic field for the NPs calcined at 700 °C and 1100 °C. The calcination temperature greatly influences the morphology and the phase constitution of the samples, and this has an important effect on the magnetic properties of the prepared compounds [26]. The hysteresis loops indicate low coercivity (*Hc*) values suggesting that the coalescence of the crystallites results in enhanced magnetic coupling and higher magnetization [16]. The *H_C_* of the spinel nano-ferrites is mainly dictated by the magnetocrystalline anisotropy, particle-particle interaction, strain, morphology and the grain sizes [27]. The *M_S_*, as well as the magnetic anisotropy of the NPs calcined at 700 and 1100 °C, increase with increasing of the Zn_0.6_Mn_0.4_Fe_2_O_4_ content in the SiO_2_ matrix.

With increasing calcination temperature, the NPs have a softer magnetic behavior since higher magnetic field are needed to fully saturate the magnetization of the samples [28]. Generally, the magnetic parameters (*M_S_* and *M_R_*) of the NPs improve with increasing calcination temperature since the crystallinity of Zn_0.6_Mn_0.4_Fe_2_O_4_ phase increases, the interatomic distances are larger, more vacancies could appear and the coordination number could diminish [13]. Besides the dilution of the magnetic matrix, the SiO_2_ matrix generates surface disorder effect on the particles, it can increase the number of broken chemical bond, leading to spin canting, pinning of the magnetic field and change of the particle size distribution [13]. As known, the cation distribution in the spinel lattice has a strong influence on the magnitude of the saturation magnetization [13,28].

The NPs calcined at 700 °C have low *M_S_* values due to their low crystallinity degree, high defect concentration, low coordination number and large interatomic spacing [13,19]. The local defects lead to the weakening of the superexchange interaction between the A and B sites [13]. The bulk density of the samples, as well as the grain sizes also can have an important effect on the magnetic behavior of the samples. The release of tension by the bigger grains is higher than by the smaller grains leading to lattice expansion [29]. The pores can act as pinning centers for the magnetic moments and for the domain walls [12]. The *H_C_* increases for the NPs calcined at 700 °C and decreases for the NPs calcined at 1100 °C, with increasing the ferrite content in the SiO_2_ matrix. The low *Hc*, values for the NPs with δ = 25, 50% calcined at 700 °C, indicating that these samples can be easily demagnetized in technical applications [12]. The higher *H_C_* values are related with Zn atoms, while the presence of Mn atoms in the ferrite results in a decrease of the coercive field [16,27,28]. The variation of the coercivity *H_c_* with increasing Zn-Mn ferrite content in the SiO_2_ matrix is related with the change of the anisotropy constant, particle size distribution and with the magnetic domains structure of the NPs [30]. The above mentioned, superparamagnetic-like behavior of these NPs arises from the small crystallite sizes with low anisotropy which are easily thermal activated [13,16,19]. As known, in polycrystalline ferrites, the variation of the main magnetic parameters (i.e., *M_S_*, *M_R_* and *H_C_*) are related with the grain sizes, bulk density, magnetic anisotropy, superexchange interaction between A and B sites and the surface effects [12].

The magnetization of MnFe_2_O_4_ is mainly determined by the competition between A-B super exchange and B-B exchange [31]. When Zn^2+^ is added in the Mn ferrite structure, the A-B exchange in weakened and B–B sublattice interaction becomes stronger. In case of NPs (δ = 75 and 100%), the presence of the α-Fe_2_O_3_ as secondary phase contributes to the overall magnetization. The *M_S_* increases with increasing content of ferrite and α-Fe_2_O_3_ phases. The decrease of magnetization can be the consequence of the spin canting in the B sublattice as described by the non-collinear Yafet-Kittel model [18,31,32,33]. At the particles surface a dead layer is formed which contains broken chemical bonds, deviations from the bulk cation distribution, non-saturation effects, randomly oriented magnetic moments, lattice defects, etc. which usually result in the depreciation of the magnetic properties of the NPs [34]. The large grains contain a great number of magnetic domains walls and the motions of these walls will have a dominant contribution to the magnetization process versus the rotation of the magnetic moments [13,16,19]. As the particle size decreases multiple domains are converted into single larger domains and align along the direction of applied field. As the motion of domain walls is responsible for reversing the field, the decrease of particle size may transform a magnetic material from multi domain phase to a single domain phase resulting in the *H_c_* increase [18].

The anisotropy constant *K* was calculated using Equation (1) [35]:

(1) $$K=\frac{\mu_{0}\cdot M_{S}\cdot H_{c}}{2}$$

where *M_S_* is the saturation magnetization, µo is the magnetic permeability of the free space (μ_0_ = 1.256 × 10^−6^ N/A^2^) and *Hc* is the coercivity field (T).

**Figure 5:**
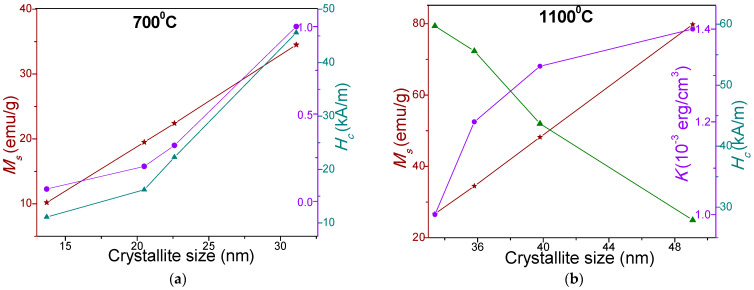
Variation of *M_S_*, *H_C_* and *K* with crystallite size of NPs calcined at 700 °C (**a**) and 1100 °C (**b**).

The linear variation of the *M_S_* as a function of the crystallite size for the NPs calcined at 700 and 1100 °C is presented in Figure 5. A non-linear increase of the coercivity (*H_C_*) (NPs calcined at 700 °C) and magnetic anisotropy constant (*K*) (NPs calcined at 700 and 1000 °C) and decrease of the *H_C_* (NPs calcined at 1100 °C), with the increase of crystallite size is observed. The behavior of *H_C_* suggests single magnetic domains for the NPs calcined at 700 °C as *H_C_* increases with increasing average particle size and multidomain regime for the NPs calcined at 1100 °C as the *H_C_* decreases with increasing average particle size. The reduction of the *H_C_* with increasing ferrite content can be connected with the particle sizes.

The TEM and XRD data indicates an increase of the particle and crystallites sizes with increasing ferrite content embedded in the SiO_2_ matrix. Similarly, an increase of the *K* with increasing ferrite content embedded in the SiO_2_ matrix was found. Therefore, we concluded that the increase of the particle sizes leads to the enhancement of the *K*. When the size of a magnetic particle increases above a critical crystallite diameter, a multidomain region occurs, where the *H_C_* reduces its value [36]. The SiO_2_ matrix generates stress on the surface of the ferrite particle, which will hamper the rotation of the magnetic moments from the dead layer at the surface contributing in this way to the reduction of the coercivity [13]. The magnetic anisotropy constant depends on the lattice crystalline symmetry, the crystalline anisotropy, and on particles size and shape [13,27]. The magnetocrystalline anisotropy is also affected by the distribution of the magnetic ions on the surface of the nanosized particles. In our case, the Mn^2+^ and Zn^2+^ ions have considerable contributions to the magnetocrystalline anisotropy. The magnetocrystalline energy enhances with increasing particle sizes and the volumes of NPs [12]. The highest *K* value is obtained for the Zn_0.6_Mn_0.4_Fe_2_O_4_ NPs (δ = 100%) for which a high magnetic field is necessary to saturate the magnetization, due to the magnetic ions disorder generated by the surface effects in the dead layer [13].

The Mn-Zn ferrite embedding in SiO_2_ matrix allows an easy control of the crystallization temperature, nanoparticle sizes and magnetic properties of the NPs. The easily tunable magnetic and electrical properties of the NPs recommend them for application as ferrofluids, hybrid supercapacitors, biocompatible magnetic-fluids, medical applications or as efficient magnetically recyclable material for the removal of chemical and biological contaminants from industrial wastewaters [37,38,39,40,41,42,43].

## 4. Conclusions

(Zn_0.6_Mn_0.4_Fe_2_O_4_)_δ_(SiO_2_)_100−δ_ (δ = 25–100%) NPs with different morphologies, phase constitutions and magnetic properties were obtained using sol-gel method. The stoichiometric composition, synthesis technique and particle size play a critical role in defining the ferrite properties. At low calcination temperatures (400 and 700 °C), single crystalline phase was obtained, excepting NC with δ = 75% at 700 °C, where the main phase was accompanied by the secondary α-Fe_2_O_3_ phase. At high calcination temperatures (1100 °C), cristobalite and quartz phases were also present. The average crystallites size increases with increasing calcination temperature, as well as with increasing ferrite content embedded in the SiO_2_ matrix: 16.15 ± 10.85 nm (400 °C), 22.4 ± 8.7 nm (700 °C) și 41.25 ± 7.85 nm (1100 °C). The TEM images show irregular aggregates of spherical, large NPs for samples with high ferrite content (50 nm) or small NPs for samples with high SiO_2_ content (34 nm) NPs. The saturation magnetization (*M_S_*) and anisotropy constant (*K*) of the NPs calcined at 700 °C (10.2–34.5 emu/g and 0.07·10^−3^–1.00·10^−3^ erg/cm^3^) and at 1100 °C (26.7–79.8 emu/g and 1.00·10^−3^–1.4·10^−3^ erg/cm^3^) increase with increasing ferrite content embedded in the SiO_2_ matrix. The coercivity field (*H_C_*) increases (11.1–45.6 kA/m) for the NPs calcined at 700 °C and decreases (59.7–27.9 kA/m) for the NPs calcined at 1100 °C with increasing ferrite content embedded in the SiO_2_ matrix. It was found a linear dependency of the *M_S_* on the crystallite’s sizes for the both calcination temperatures (700 and 1000 °C).

## Figures and Tables

**Table 1:** The crystallite size and quantitative analysis according to XRD of (Mn_0.6_Mn_0.4_Fe_2_O_4_)_δ_(SiO_2_)_100−δ_ NPs calcined at 400, 700 and 1100 °C.

Sample	Temperature (°C)	D (nm)	Quantitative Analysis (%)
δ = 25%	400	5.3	100% Zn_0.6_Mn_0.4_Fe_2_O_4_
	700	13.7	100% Zn_0.6_Mn_0.4_Fe_2_O_4_
	1100	33.4	52% Zn_0.6_Mn_0.4_Fe_2_O_4_/48% SiO_2_
δ = 50%	400	6.7	100% Zn_0.6_Mn_0.4_Fe_2_O_4_
	700	20.5	100% Zn_0.6_Mn_0.4_Fe_2_O_4_
	1100	34.5	59% Zn_0.6_Mn_0.4_Fe_2_O_4_/41% SiO_2_
δ = 75%	400	7.8	100% Zn_0.6_Mn_0.4_Fe_2_O_4_
	700	22.6	89% Zn_0.6_Mn_0.4_Fe_2_O_4_/11% α-Fe_2_O_3_
	1100	39.8	71% Zn_0.6_Mn_0.4_Fe_2_O_4_/10% α-Fe_2_O_3_/19% SiO_2_
δ = 100%	400	27.0	46% Zn_0.6_Mn_0.4_Fe_2_O_4_/44% α-Fe_2_O_3_/10% ZnO
	700	31.1	49% Zn_0.6_Mn_0.4_Fe_2_O_4_/45% α-Fe_2_O_3_/6% ZnO
	1100	49.1	73% Zn_0.6_Mn_0.4_Fe_2_O_4_/27% α-Fe_2_O_3_
